# Variant genotyping with gap filling

**DOI:** 10.1371/journal.pone.0184608

**Published:** 2017-09-08

**Authors:** Riku Walve, Leena Salmela, Veli Mäkinen

**Affiliations:** Helsinki Institute for Information Technology HIIT, Department of Computer Science, University of Helsinki, Helsinki, Finland; University Hospital Jena, GERMANY

## Abstract

Although recent developments in DNA sequencing have allowed for great leaps in both the quality and quantity of genome assembly projects, *de novo* assemblies still lack the efficiency and accuracy required for studying genetic variation of individuals. Thus, efficient and accurate methods for calling and genotyping genetic variants are fundamental to studying the genomes of individuals. We study the problem of genotyping insertion variants. We assume that the location of the insertion is given, and the task is to find the insertion sequence. Insertions are the hardest structural variant to genotype, because the insertion sequence must be assembled from the reads, whereas genotyping other structural variants only requires transformations of the reference genome. The current methods for constructing insertion variants are mostly linked to variation calling methods and are only able to construct small insertions. A sub-problem in genome assembly, the gap filling problem, provides techniques that are readily applicable to insertion genotyping. Gap filling takes the context and length of a missing sequence in a genome assembly and attempts to assemble the intervening sequence. In this paper we show how tools and methods for gap filling can be used to assemble insertion variants by modeling the problem of insertion genotyping as filling gaps in the reference genome. We further give a general read filtering scheme to make the method scalable to large data sets. Our results show that gap filling methods are competitive against insertion genotyping tools. We further show that read filtering improves performance of insertion genotyping especially for long insertions. Our experiments show that on long insertions the new proposed method is the most accurate one, whereas on short insertions it has comparable performance as compared against existing tools.

## Introduction

High-throughput sequencing is today part of the standard toolbox in life science research. However, despite the advances in sequencing technologies fully constructing the genome of an individual, i.e. *de novo* genome assembly, is still a time consuming task especially for large eukaryotic genomes [1]. Thus if a reference genome is available, like for the human genome, usually a resequencing approach is applied to determine the genetic variants in a donor genome as compared to the reference. In a resequencing project, the donor genome is first sequenced, the sequencing reads are then aligned against the reference genome, and finally the genetic variants are inferred based on the aligned reads [2].

Genetic variants are generally split into two groups based on their size. In *single nucleotide polymorphisms*, *SNPs*, a single nucleotide of the donor genome differs from the reference sequence. Larger variants are called *structural variants* and include for example deletions, inversions, insertions, and duplications. The problems of finding the positions of structural variants and *genotyping*, i.e. determining the actual variants, are two different problems although in many tools methods to solve them are intertwined and most tools do their best at answering both questions.

In this paper we will address the insertion genotyping problem. We are given the position of an insertion in a reference genome and a set of sequencing reads from a donor genome. We can now infer the sequences flanking the insertion based on the reference genome and the length of the insertion can be inferred based on paired end sequencing reads. The insertion genotyping problem is then to use the sequencing reads to infer an insertion sequence of correct length that bridges the gap between the flanking sequences. We see that the insertion genotyping problem resembles *de novo* genome assembly, especially when the length of the insertion grows since the task is to infer the part of the sequence not present in the reference genome.

A similar problem is faced in the last phase of *de novo* genome assembly, gap filling. *Gap filling* is the problem of reconstructing the missing sequence between contiguous sections, called *contigs*, of an assembly that have a gap of either an estimated or an unknown length between them. Also in this case the flanks of the missing sequence are known and the task is to infer the missing sequence given the sequencing reads and an estimate of the length of the sequence. In gap filling, the gaps are sections that have proved difficult to assemble. The difficulty arises mainly from two sources, either the section has been sequenced with a low coverage or it contains too much repetitive sequences to unambiguously assemble.

Many genome assemblers, such as Allpaths-LG [3] and ABySS [4], include a gap filling module in their pipelines. There are also standalone gap filling tools available, e.g. SOAPdenovo’s GapCloser [5], GapFiller [6], Gap2Seq [7], MindTheGap [8] and Sealer [9]. All these tools attempt to do *local genome assembly* with a set of reads from the genome but the actual methods used vary. Allpaths-LG uses overlaps within a subset of the reads and GapFiller uses a *k*-mer based method. GapCloser, Gap2Seq, MindTheGap, and Sealer use graph based methods.

In this paper we extend the definition of gap filling to the insertion genotyping problem and modify a gap filling tool, Gap2Seq [7], into an insertion genotyping tool. Among the gap filling tools, Gap2Seq has a unique feature of respecting the length of the gap, making it a robust option as a basis for insertion genotyping. We further introduce and implement a general read filtering scheme to make Gap2Seq scale to large data sets used in resequencing projects and show how it can be applied to insertion genotyping. We investigate experimentally the applicability of gap fillers to the insertion genotyping problem and compare them to tools developed for insertion genotyping. Our results show that insertion genotyping utilizing the insertion length estimate improves the accuracy of insertion genotyping on long insertions significantly.

## Methods

### Gap filling

*De novo* genome assembly attempts to reconstruct the genome of a species based on a set of sequencing reads *R*. In a typical pipeline the reads are first joined into *contigs* which are contiguous sections of the target sequence. Contigs are then ordered into *scaffolds* and finally the gaps between consecutive contigs are filled by reusing the sequencing reads.

Contig assembly is often abstracted as the problem of reconstructing a string from a set of its *k*-mers which can be done using *de Bruijn graphs*. A *k*-th order de Bruijn graph is a directed graph *G* = (*V*, *E*) where vertices *v* ∈ *V* correspond to *k*-mers present in the set of reads and edges (*v*, *v*′) ∈ *E* correspond to observed (*k* + 1)-mers in the reads starting with *v* and ending with *v*′.

Gap filling is the process of reconstructing the missing sequence between consecutive contigs that have a gap of either an estimated or an unknown length between them. Salmela et al. [7] formulate the problem as a modified *exact path length problem*, which we will call the path length problem here.

**Definition 1.** *Path Length problem*. Given a directed graph *G* = (*V*, *E*), two vertices *s*, *t* ∈ *V*, a cost function $c:E\to Z_{\geq 0}$, and an interval of path costs [*d*′..*d*], find a path *P* = *v*_1_*v*_2_ ⋯ *v*_*n*_ such that *v*_1_ = *s*, *v*_*n*_ = *t*, and

$$\begin{array}{c} \mathrm{Cost}\left(P\right)=\sum_{i=1}^{n-1}c(v_{1},v_{i+1})\in \left[d^{\prime }..d\right]. \end{array}$$

The problem was shown to be solvable efficiently with simple dynamic programming [7]. We now give a brief overview of the solution and refer the interested reader to the earlier paper.

We fill a matrix *M*[*v*][*i*], where *v* ∈ *V* and *i* ∈ [1..*d*], such that *M*[*v*][*i*] is the number of paths that reach *v* from *s* and the cost of the path is exactly *i*. This can be done with a breadth-first search in *G* where we increment *M*[*v*][*i*] when we reach *v* from any vertices *w* where *M*[*w*][*i* − *c*(*w*, *v*)] > 0. We then trace back any path *P* = *s* ⋯ *t* where *M*[*t*][*i*] > 0 for any *i* ∈ [*d*′..*d*] and output the labels of the vertices in the path.

Given the de Bruijn graph used for genome assembly, we can trivially define the cost function to be 1 for every edge in the graph, i.e. *c*(*v*, *w*) = 1 if and only if (*v*, *w*) ∈ *E*. Now, as the overlap of connected vertices is always *k* − 1, the cost for a path is its length.

**Definition 2.** *Gap Filling problem*. Given a *k*-th order de Bruijn graph *G* = (*V*, *E*), two *k*-mers *s*, *t* ∈ *V*, and an estimated gap length *d*, find a path *P* = *s* ⋯ *t* such that the length of the path is close to *d*.

Using this definition, we can use the solution for the path length problem to find any acceptable solution for the gap filling problem. We only need to consider how to construct the interval [*d*′..*d*] from the estimated gap length. Salmela et al. suggest using an interval, where the midpoint (*d*′ + *d*)/2 is the estimated gap length.

As the sequences around the gap are likely to have assembly errors, allowing the paths to start and end beyond the exact borders of the gap is a simple way to get rid of the errors. GapFiller [6] accomplishes this by simply removing the flanking sequences from the assemblies. Gap2Seq [7] uses a more conservative approach by looking at the paths that start and end at different points in the flanking sequences.

### Read filtering

When filling a gap, we would intuitively want to only use the subset of reads that cover the given gap. By restricting the set of reads to those that cover the gap, we can give stricter assumptions about the distribution of the *k*-mers. To find all the reads that cover a region of the scaffold, we will use *read filtering*.

The read filtering problem is defined formally as follows.

**Definition 3.** *Read Filtering problem*. Given an interval of a gap [*s*..*e*] and read alignments *A*(*r*) = [*s*_*r*_..*e*_*r*_] for reads *r* ∈ *R*, find reads *r* ∈ *R* that overlap with the gap.

If all reads were aligned, filtering reads would be fairly trivial. However, especially with long insertions all reads do not align, as the reads would have to be aligned to parts that do not exist in the contigs.

We can instead use paired-end reads with at least one end aligned which gives an estimate for the position of the other end. Gap filling methods tackle this problem in different ways. GapFiller [6], for example, takes all the unaligned reads from read pairs with one read aligned within a maximum distance from the gap.

Finding all reads whose pair would be in a region *S* = [*s*..*e*] can also be seen as finding all the reads that map to a region that is defined symmetrically on both sides of *S* by the first and last possible positions a read could start from to have a mate belong to *S*. The regions *S*_left_ and *S*_right_ can be defined using the parameters of the original region *S*, the read length *ℓ*, and the expected insert size. The regions are defined as,

$$\begin{array}{ll} S_{\text{left}} & =\left[s-\left(\text{max}+2l\right)..e-\left(\text{min}+l\right)\right],\\ S_{\text{right}} & =\left[s+\left(\text{min}+l\right)..e+\left(\text{max}+l\right)\right], \end{array}$$

where max and min are the maximum and minimum insert sizes respectively.

The values for max and min can be computed from the distribution of insert sizes. For example, choosing the insert sizes to be within the 95% confidence interval of the distribution, i.e. *P*(|*X*| ≥ *I*) ≤ 0.05, gives us maximum and minimum insert sizes of *μ* ± 1.96*σ*, where *μ* is the average insert size and *σ* is the standard deviation of the insert size.

Note that we assume reads to be of the same length, which is often the case. The regions could also be defined by giving maximum and minimum read lengths if the read lengths fall into some distribution, such as when using long reads.

As the sequenced reads can be assumed to be read from random positions, we can further assume that all positions are sequenced with equal coverage. Thus we can calculate the expected coverage $C=\frac{\left|reads\right|}{\left|genome\right|}$ and expect the set of filtered reads to have the same coverage $\frac{\left|filtered\;reads\right|}{\left|region\right|}\approx C$. If the read filtering gives a coverage that is significantly smaller than expected, we are likely to be missing reads that are unmapped. All the unmapped reads can then be added to the set of filtered reads for better gap filling performance.

### Insertion genotyping

The insertion genotyping problem can be defined essentially the same way as the gap filling problem.

**Definition 4.** *Insertion Genotyping problem*. Given a *k*-th order de Bruijn graph *G* = (*V*, *E*), a breakpoint position *p* on the reference *R*, and an estimated length of insertion *d*, find a path *P* = *s* ⋯ *t* in *G*, such that *s* = *R*[*p* − *k*..*p*], *t* = *R*[*p*..*p* + *k*], and the length of the path is close to *d*.

Using this definition, we can use gap filling to solve insertion genotyping. The main difference is that in gap filling the unresolved gaps are in genomic regions that are hard to assemble, either because of low coverage of reads or because of repetitions, whereas insertions can simply be random sequences inserted to the genome. However, also the insertion sequences are more likely to be repeated sequences from the genome in which case the difficulty is similar to gap filling.

In practice, we can simply take a reference genome and the set of structural variations and for every insertion variant, insert a gap of the estimated length at the corresponding position in the reference genome. Filling the gaps on this sequence then constructs the donor genome.

Although overlapping variations should be taken into consideration, doing so would require accurate knowledge of all the surrounding variations. As we are interested in specifically assembling insertion variants, we will assume no other variations overlap with the flanking sequences.

Note that this assumption is related to the issue of assembly errors in the gap filling problem and similar solutions are useful here. Either completely removing the edges of the flanking sequences or letting the paths skip any parts of them are applicable.

## Results and discussion

### Read filtering

Read filtering is evaluated experimentally by generating assemblies from the reference by removing sequences of varying lengths. Reads are then generated from the full reference sequence and mapped to the assemblies and filtered based on the alignments. The filtering is compared to a known truth by mapping the same reads to the reference genome without the gaps and taking all the reads that overlap with a given gap.

The read filtering results can be partitioned into four groups: true positives, reads correctly filtered in; false positives, reads incorrectly filtered in; true negatives, reads correctly filtered out; and false negatives, reads incorrectly filtered out. We then use the metrics *precision* and *recall* to evaluate the filtering scheme:

$$\begin{array}{cc} \text{Precision} & =\frac{\text{true}\;\text{positives}}{\text{true}\;\text{positives}+\text{false}\;\text{positives}},\\ \text{Recall} & =\frac{\text{true}\;\text{positives}}{\text{true}\;\text{positives}+\text{false}\;\text{negatives}}. \end{array}$$

The reference genome used to generate reads is chromosome 17 from the version GRCH38 of the human reference genome. The ambiguous bases in the sequence are replaced by random nucleotides, as in the gap filling context they are used to mark gaps in the sequences.

Paired-end reads were generated with ART [10] from the full reference sequence. The simulated reads have a read length of 100 bp and a coverage of 30x. For evaluating the read filtering the threshold for using unmapped reads was set to 25. It should be set close to the coverage of the read libraries, though we will discuss this later in this section. For Gap2Seq, all de Bruijn graphs were built with a fixed *k* of 31, whereas Sealer uses multiple values of *k* and was run with *k* 28 through 34. Other tools used default settings.

We generated three read sets with different insert size distributions. Mean insert sizes for the read sets were 150 bp, 1500 bp, and 3000 bp, and standard deviations were 15, 150, and 300, respectively. These parameters are motivated by the read libraries in the GAGE data sets [11].

To simulate insertion genotyping, for each run of the experiments, we randomly inserted 100 insertions into the reference with lengths randomly chosen between 10 and 1000 bp. The experiments were then averaged over 5 runs. All the scripts used in the simulations can be found at https://github.com/rikuu/eval-insertfill/.

We evaluated three read filtering schemes:

- *Unmapped*: All unmapped reads are filtered in.
- *Filter*: Filtering based on insert size as described in the previous section.
- *GapFiller*: The filtering used in GapFiller. I.e. pairs of all unmapped reads whose pair aligns within a maximum distance from the gap are filtered in.

**Fig 1:**
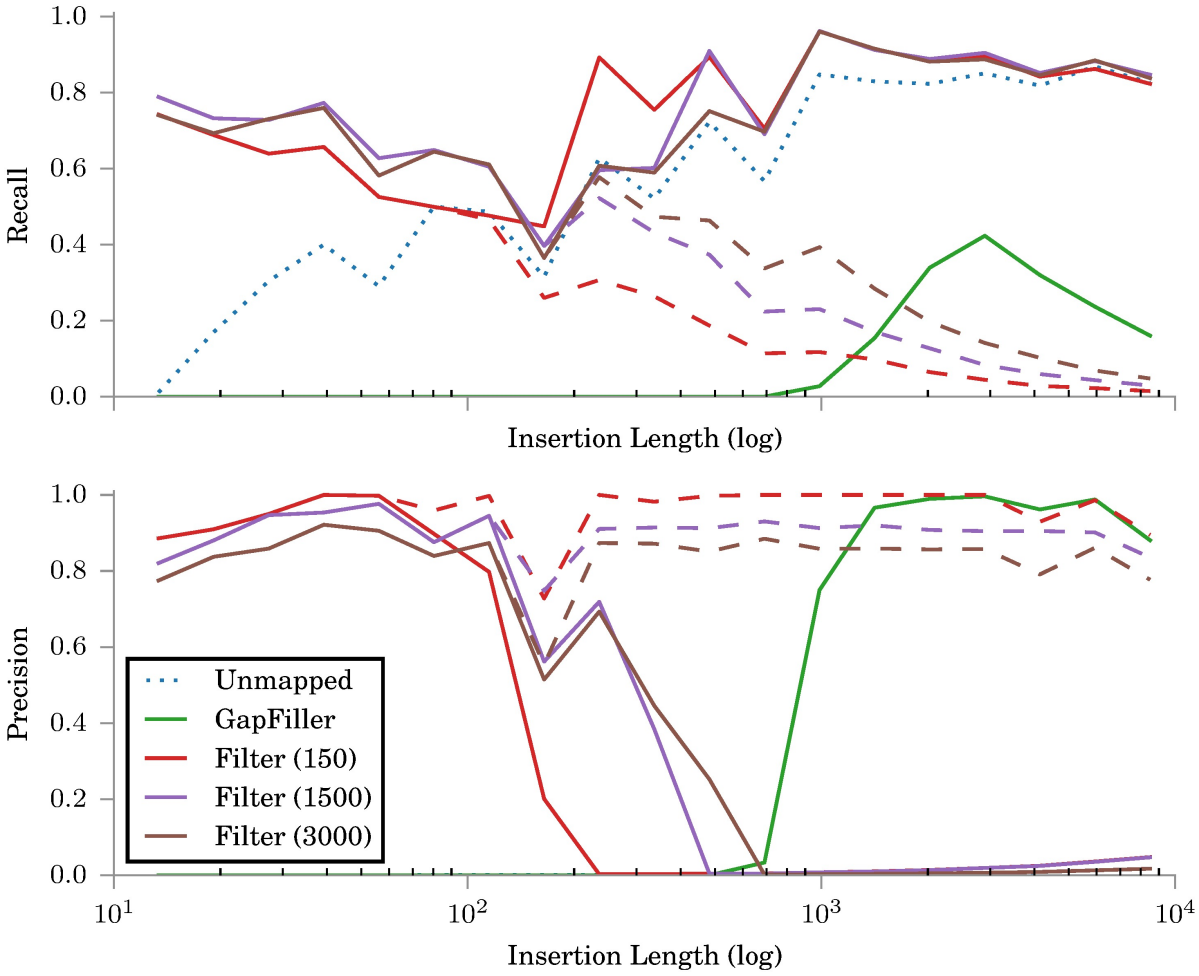
Recall (a) and precision (b) scores for simulated insertions. Filtering is tested with three simulated read sets with different insert size distributions. As the results for our proposed filtering scheme differ based on the insert size distributions, we have separated and labeled them with the corresponding mean insert sizes, *μ* = 150, 1500, 3000. The other two are averaged over all the read sets. The dashed lines for Filter use only reads that are filtered in and the solid lines add all unmapped reads to the filtered read set if the coverage of the filtered reads is below the coverage threshold (here 25). The precision of using all unmapped reads is almost zero and is thus not visible in the graph.

The recall scores in Fig 1a show that estimating read alignments by paired-end read pairs is useful up to a point. This method fails to find reads from a growing section in the middle of the gap when the gap length exceeds the insert size and no reads can be estimated to cover the middle of the gap.

The recall scores for different insert sizes shows that larger insert sizes generally give better filtering results than smaller insert sizes. However, increasing the mean insert size in practice also increases the standard deviation of the insert size distribution. Thus it also affects the quality of the read pair alignment position estimation.

The precision scores in Fig 1b show the negative effect of using the unmapped reads in addition to the filtered reads. Due to the fact that the unmapped reads can originate from any insertions, the unmapped reads always give a low precision score.

Finding a good threshold for using the unmapped reads means finding a balance between either having too few reads to find a useful path over the gap or having too many reads to find paths in the graph. That said, we found no meaningful differences between its exact values, only that it should be close to but smaller than the coverage of the read libraries.

### Insertion genotyping on simulated data

To evaluate the quality of insertion genotyping, we use the normalized edit distance as a score throughout. The edit distance *ed*(*S*_1_, *S*_2_) of two sequences *S*_1_ and *S*_2_ is the minimum number of substitutions, insertions and deletions needed to transform one sequence into the other. We further divide the edit distance by the length of the true sequence to make the results between gap lengths comparable. The score is thus defined as follows:

$$\text{Score}\left(\text{output},\text{correct}\right)=\frac{ed(\text{output},\text{correct})}{\left|\text{correct}\right|}.$$

The perfect score of 0 is achieved with an output that is exactly the correct insertion. A score of 1 means that either the insertion was not genotyped, or that the insertion was genotyped and of the correct length but entirely incorrect content.

**Fig 2:**
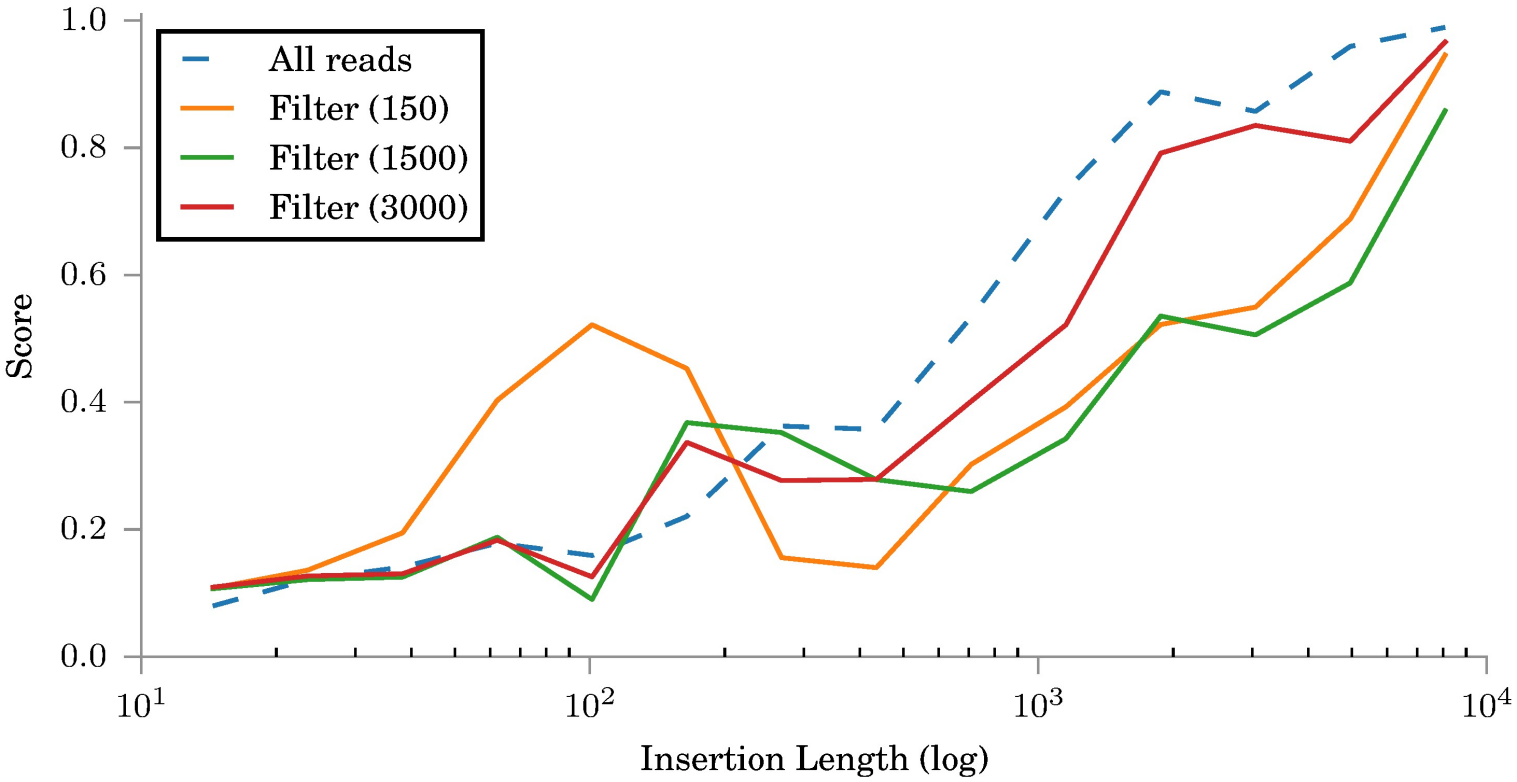
Scores for simulated insertions. Filtering is simulated with three simulated read sets with different insert size distributions. The filter schemes are labeled with the corresponding mean insert sizes, *μ* = 150, 1500, 3000.

To evaluate insertion genotyping we used the same simulated data sets as for evaluating read filtering. Fig 2 shows how the read filtering affects the quality of insertion genotyping. The read filtering should generally perform better with sequencing libraries that have a large insert size but the effect is not very pronounced. This could be due to the fact that the unmapped reads are added when the coverage of the reads filtered in is low.

Using all available reads is better at accurately constructing smaller insertions. However, with long insertions the insertion sequences are often not found when using all reads due to the complex graph structure and thus read filtering becomes a requirement for successful insertion genotyping. However, even with read filtering insertion genotyping is not perfect.

Of the three simulated read libraries, the middle one with *μ* = 1500 gives the best result, most likely due to it having both a large insert size and only a modest standard deviation giving a reliable filtering. We used only this read library when running a comparison of different insertion genotyping tools.

**Fig 3:**
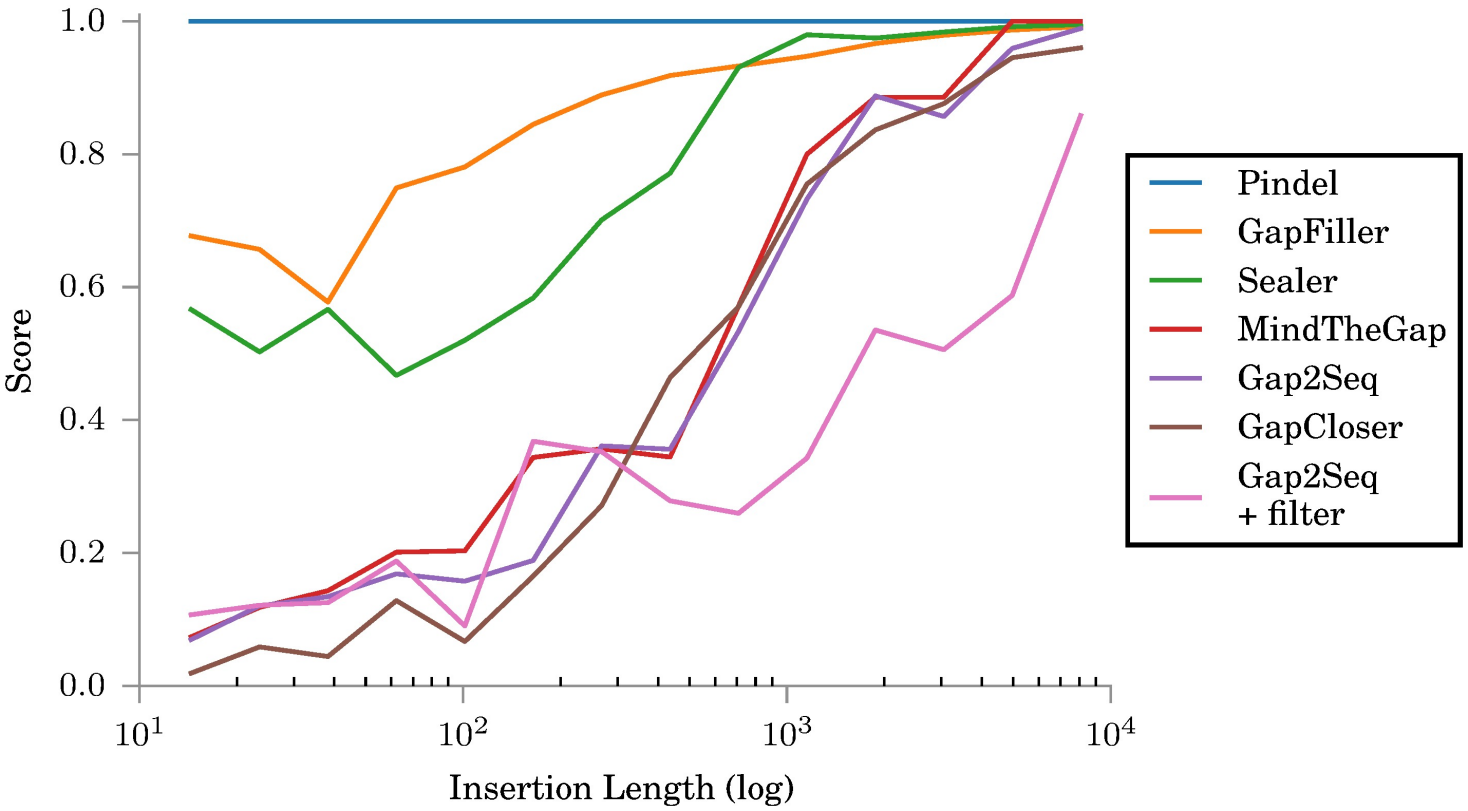
Scores for different tools on simulated insertions. All tools use the same simulated read library with *μ* = 1500.

Fig 3 shows Gap2Seq with and without filtering compared against insertion genotyping tools, Pindel [12] and MindTheGap [8], and other gap filling tools, GapFiller [6], GapCloser [5] and Sealer [9]. The positions of the simulated insertion sites were given as input for MindTheGap, GapFiller, GapCloser and Sealer. Notably, MindTheGap does not take the insertion length as part of its input, rather it outputs insertions for any length it is able to fill. Pindel does not take insertion sites as input but rather estimates the insertion sites itself. However, on this data set Pindel could not find meaningful insertions.

For short insertion lengths, Gap2Seq with filtering and GapCloser are the most accurate. For long insertions, Gap2Seq with filtering gives by far the most accurate results.

**Fig 4:**
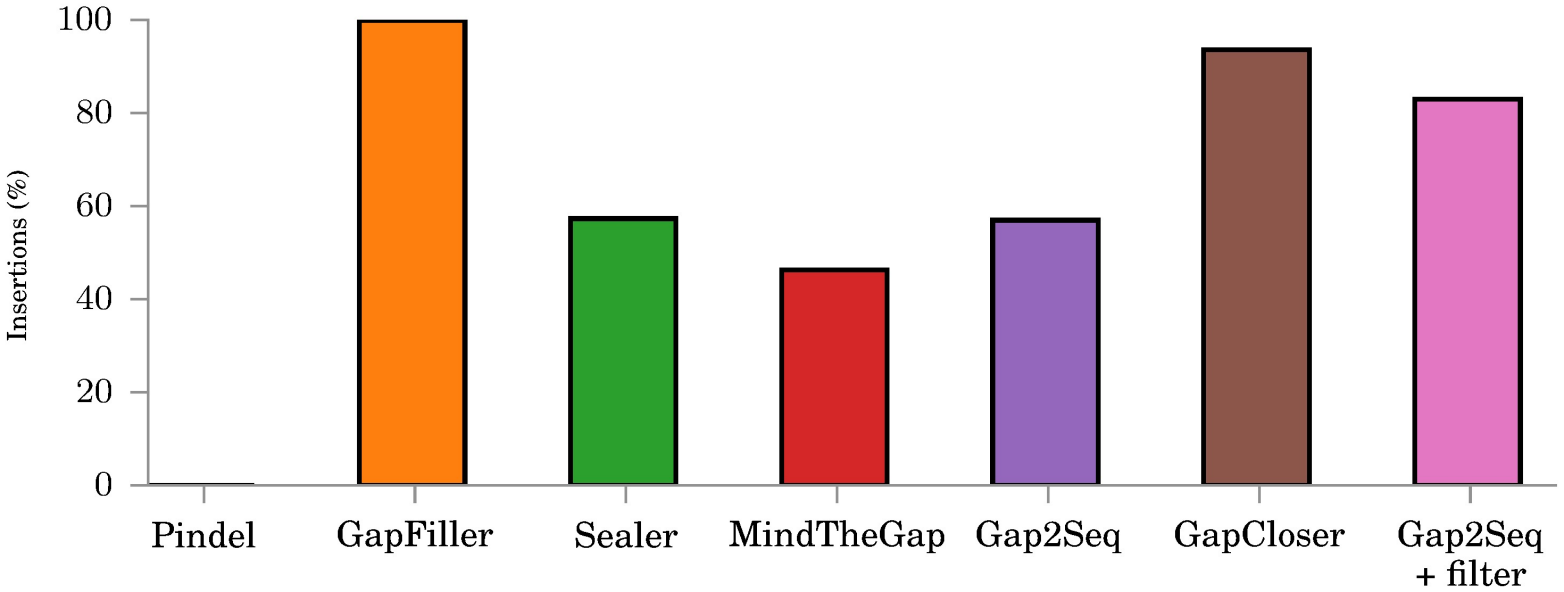
Number of insertions genotyped on the simulated data by each tool. All tools use the same simulated read library with *μ* = 1500.

Fig 4 shows how many insertions each tool is actually able to fill regardless of quality. GapFiller, GapCloser, and Sealer are able to aggressively fill almost all gaps, but as noted before, GapFiller and Sealer do not give reliable quality on any insertion lengths and also the quality of GapCloser is clearly worse than the quality of Gap2Seq with filtering for long insertions. When compared against MindTheGap and Gap2Seq without filtering, Gap2Seq with filtering is able to genotype more insertions with better quality.

**Fig 5:**
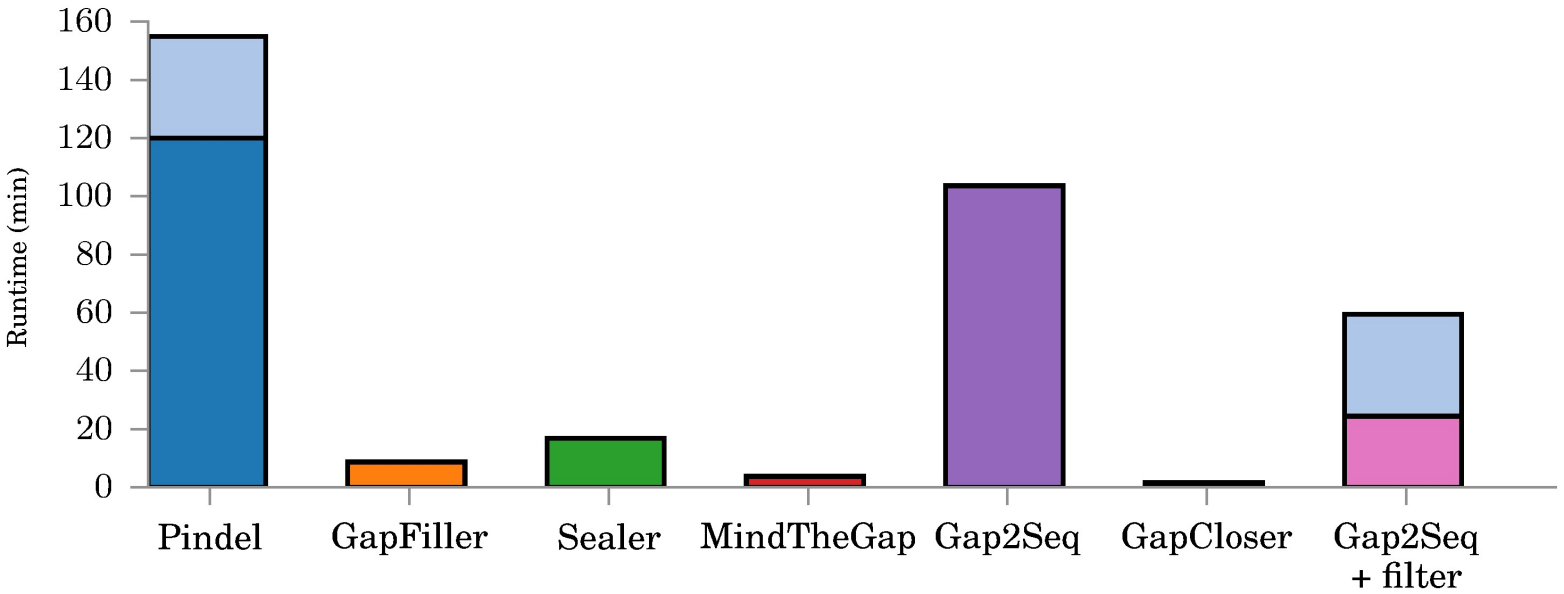
Running times for different tools on simulated insertions. For Pindel and Gap2Seq with filtering, the time to align reads to reference is shown in light blue. Note that Pindel also uses time to find the insertion sites and as such any direct comparisons are unfair.

Fig 5 shows the running times for the various tools on the simulated data. For Gap2Seq with filtering and Pindel we are separately showing the runtime of read alignment and the runtime of actual insertion genotyping (as the light blue bars on top of the runtimes of the tools). We note that usually insertion genotyping is performed along with e.g. SNP calling and thus read alignments are readily available and do not need to be rerun separately for insertion genotyping.

In the above experiments the coverage of the read set was always set to 30x. We also ran experiments with coverage 15x and 50x. In Gap2Seq with filtering the threshold for including all unmapped reads depends on the coverage of the data. As mentioned earlier, the threshold should be less than the coverage, yet somewhat close to it. Here we used a threshold of 10 for 15x data and 45 for 50x data. Otherwise the same parameters were used when running the programs.

**Fig 6:**
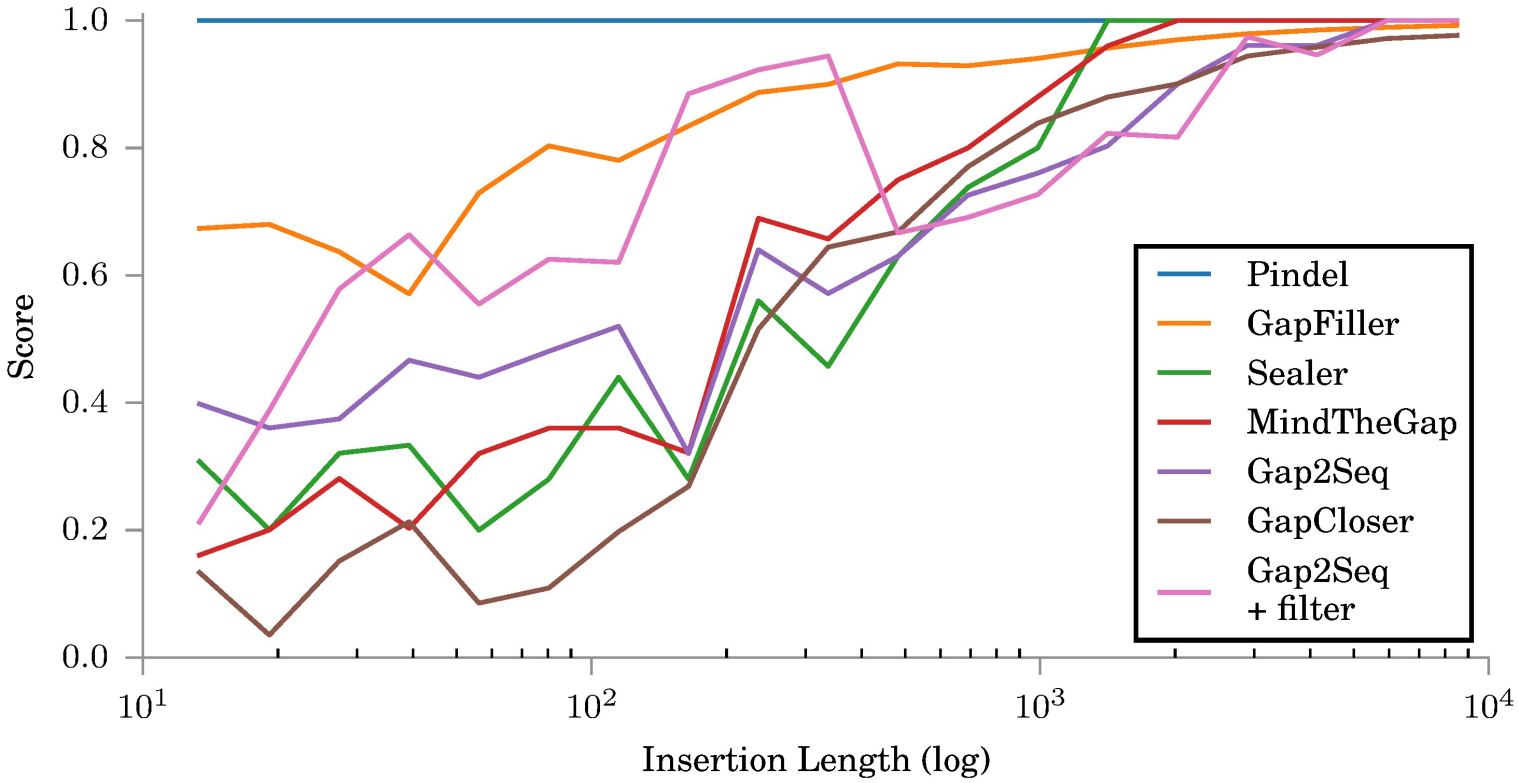
Scores for different tools on simulated insertions with 15x data. All tools use the same simulated read library with *μ* = 1500.

**Fig 7:**
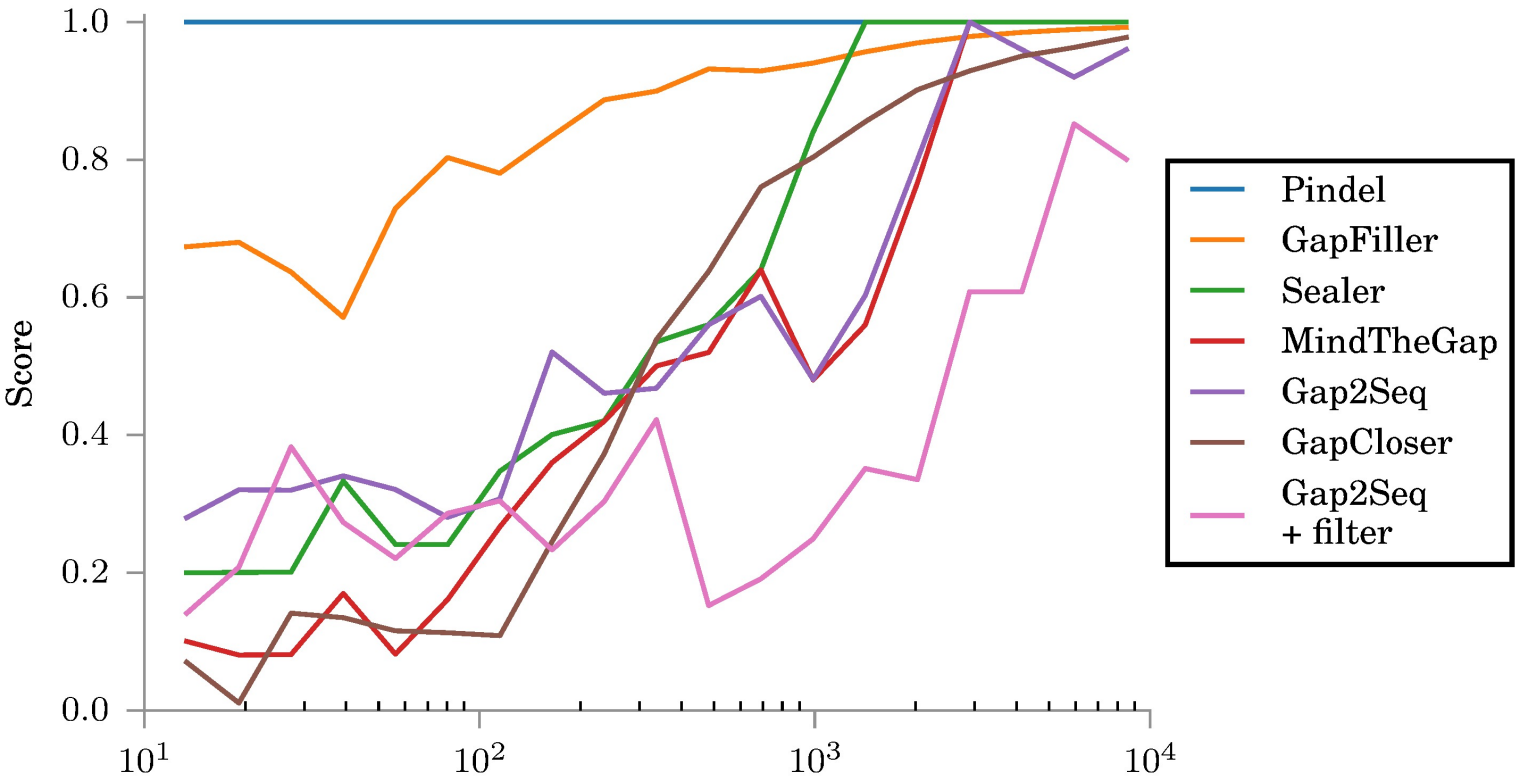
Scores for different tools on simulated insertions with 50x data. All tools use the same simulated read library with *μ* = 1500.

The results of these experiments are shown in Figs 6 and 7. We see that on long insertions the results are similar regardless of coverage but some differences can be seen on short insertions. Additionally we note that the performance of Gap2Seq with filtering suffers from low coverage because erroneously filtering out reads can easier lead to completely missing a part of the insertion when the coverage is low. However, even with the low coverage data, Gap2Seq with filtering is the best method for long insertions.

As we noted earlier, we have not found the threshold for including unmapped reads to actually affect the results of the filtering significantly. As such, rather than looking for good parameters, we recommend solving these problematic cases by using Gap2Seq without filtering with low coverage and short insertions.

### Biological data

The reference genome in the biological data experiments for insertion genotyping is the WS210 version of the *C. elegans* genome. The donor genome used is the *C. elegans* Hawaiian strain CB4856, more specifically the recent Illumina sequencing SRX523826.

The insertions were evaluated using experimentally validated insertions [13]. We used insertion sites found by Pindel [12], and Breakdancer [14]. Insertion breakpoints found by MindTheGap were not used, as they do not have the corresponding length information for the insertions. However, the combined insertion sites from the aforementioned tools were separately given as input to MindTheGap.

**Fig 8:**
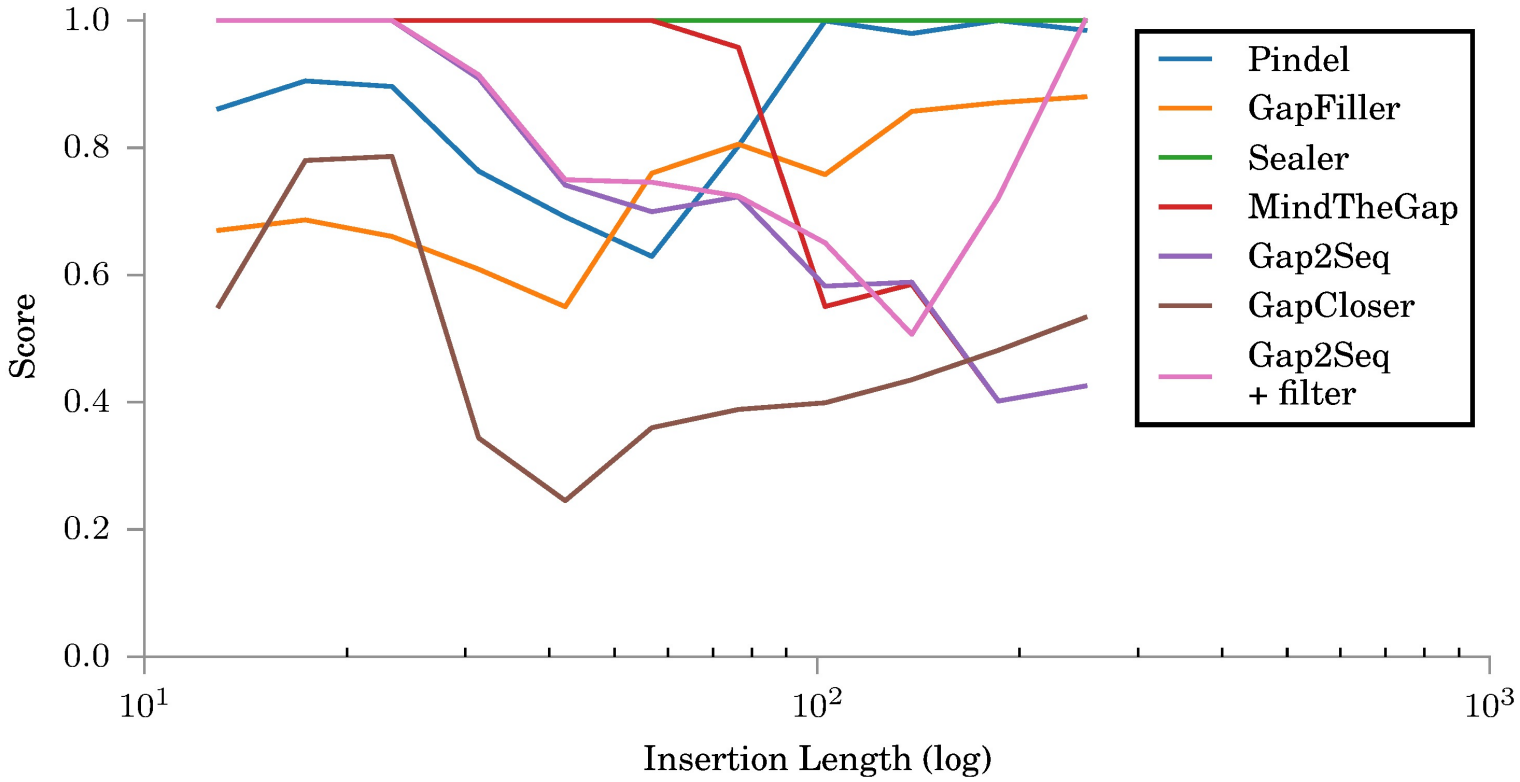
Scores for insertions constructed by different tools against validated insertions.

Gap2Seq with and without filtering was again compared against insertion genotyping tools, Pindel [12] and MindTheGap [8], and other gap filling tools, GapFiller [6], and Sealer [9]. Fig 8 shows that for short insertions, GapCloser is the most accurate tool, whereas for longer insertions also Gap2Seq and MindTheGap are competitive. The insertions in this data set were short. Based on the experiments on the simulated data, we expect Gap2Seq with filtering to be competitive for longer insertions.

**Fig 9:**
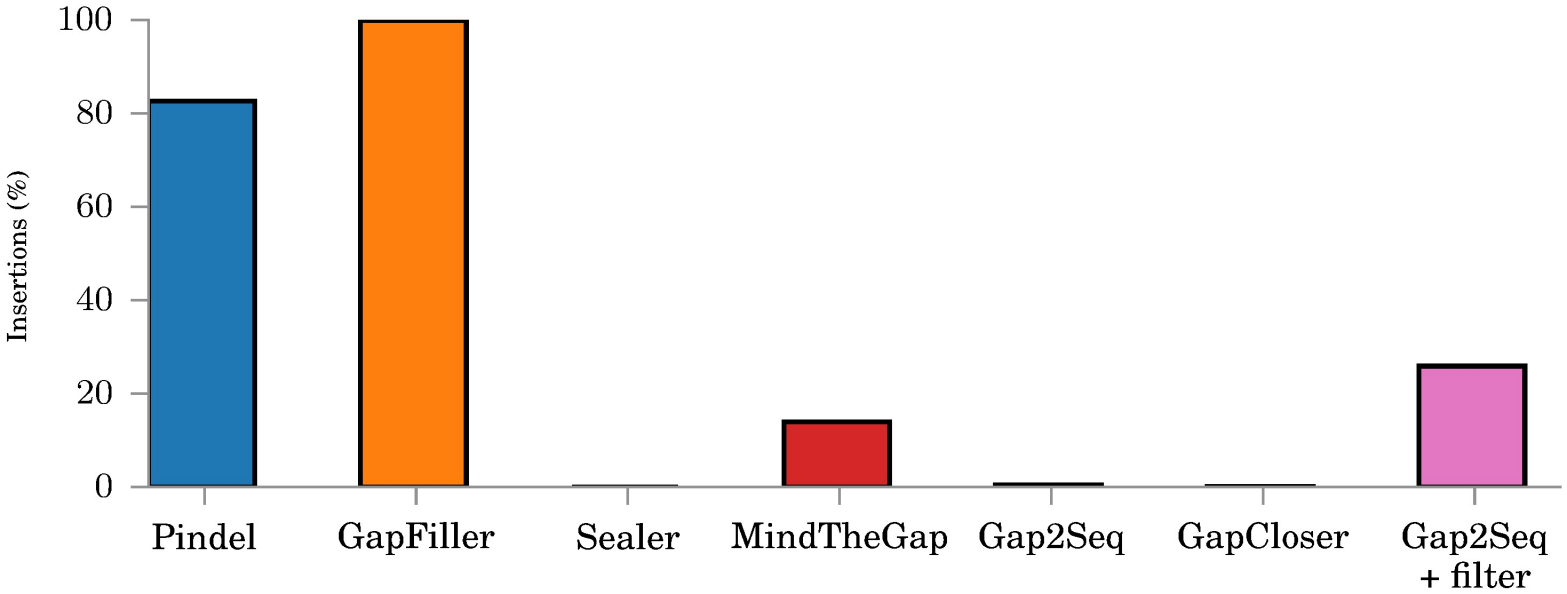
The numbers of insertions genotyped by each tool from the set of insertion sites by Breakdancer and Pindel.

Fig 9 shows how many of the insertions are genotyped by each tool. Although GapFiller and Pindel are able to construct a large number of insertions, the previous results would imply they are aggressively generating low quality sequences.

## Conclusions

We have shown how gap filling tools developed for *de novo* genome assembly can be applied to the insertion genotyping problem and how the performance of these tools can be improved using read filtering. We note that read filtering can also be useful to boost the performance of gap filling in the *de novo* assembly setting. We showed that our gap filling tool achieves comparable accuracy on short insertions and better accuracy than previous tools on insertion genotyping for long insertions which we believe to be due to read filtering and using the insertion length estimate in a rigorous way.

Last we note that our gap filling tool Gap2Seq has recently been improved with classifying the filled sequence into safe and unsafe bases where safe bases are those that are present in *any* filling sequence of correct length that can be inferred from the read set [15]. We expect this feature to be very useful in the insertion genotyping context because it provides information on the quality of the bases in the returned insertion sequence.

Our method for insertion genotyping, Gap2Seq 3.0, is freely available at https://github.com/rikuu/Gap2Seq/.

## References

[1] Bradnam KR, Fass JN, Alexandrov A, Baranay P, Bechner M, Birol I, et al Assemblathon 2: evaluating de novo methods of genome assembly in three vertebrate species. GigaScience. 2013 7 10.1186/2047-217X-2-10 23870653 PMC3844414

[2] Nielsen R, Paul JS, Albrechtsen A, Song YS. Genotype and SNP calling from next-generation sequencing data. Nat Rev Genet. 2011 6 12:443–51. 10.1038/nrg2986 21587300 PMC3593722

[3] Gnerre S, MacCallum I, Przybylski D, Ribeiro FJ, Burton JN, Walker BJ, et al High-quality draft assemblies of mammalian genomes from massively parallel sequence data. Proc. Natl. Acad. Sci. U.S.A. 2011 1 10.1073/pnas.1017351108 21187386 PMC3029755

[4] Simpson JT, Wong K, Jackman SD, Schein JE, Jones SJ, Birol I. ABySS: a parallel assembler for short read sequence data. Genome Res. 2009 6 10.1101/gr.089532.108 19251739 PMC2694472

[5] Luo R, Liu B, Xie Y, Li Z, Huang W, Yuan J, et al SOAPdenovo2: an empirically improved memory-efficient short-read de novo assembler. GigaScience. 2012 12 10.1186/2047-217X-1-18 23587118 PMC3626529

[6] Boetzer M, Pirovano W. Toward almost closed genomes with GapFiller. Genome Biol. 2012 6 10.1186/gb-2012-13-6-r56 22731987 PMC3446322

[7] Salmela L, Sahlin K, Mäkinen V, Tomescu AI. Gap filling as exact path length problem. Journal of Computational Biology 23(5);347–361. 2016 10.1089/cmb.2015.0197 26959081

[8] Rizk G, Gouin A, Chikhi R, Lemaitre C. MindTheGap: integrated detection and assembly of short and long insertions. Bioinformatics. 2014 12 10.1093/bioinformatics/btu545 PMC4253827 25123898

[9] Paulino D, Warren RL, Vandervalk BP, Raymond A, Jackman SD, Birol I. Sealer: a scalable gap-closing application for finishing draft genomes. BMC Bioinformatics. 2015 7 10.1186/s12859-015-0663-4 26209068 PMC4515008

[10] Huang W, Li L, Myers JR, Marth GT ART: a next-generation sequencing read simulator Bioinformatics. 2011 12. 10.1093/bioinformatics/btr708 PMC3278762 22199392

[11] Salzberg SL, Phillippy AM, Zimin A, Puiu D, Magoc T, Koren S, et al GAGE: A critical evaluation of genome assemblies and assembly algorithms. Genome research, 22(3), pp.557–567. 10.1101/gr.131383.111 22147368 PMC3290791

[12] Ye K, Schulz MH, Long Q, Apweiler R, Ning Z. Pindel: a pattern growth approach to detect break points of large deletions and medium sized insertions from paired-end short reads. Bioinformatics. 2009 11 10.1093/bioinformatics/btp394 PMC2781750 19561018

[13] Vergara IA, Tarailo-Graovac M, Frech C, Wang J, Qin Z, Zhang T, et al Genome-wide variations in a natural isolate of the nematode Caenorhabditis elegans. BMC Genomics. 2014 4 10.1186/1471-2164-15-255 24694239 PMC4023591

[14] Chen K, Wallis JW, McLellan MD, Larson DE, Kalicki JM, Pohl CS, et al BreakDancer: an algorithm for high-resolution mapping of genomic structural variation. Nature Methods. 2009 8 10.1038/nmeth.1363 PMC3661775 19668202

[15] Salmela L, Tomescu AI Safely filling gaps with partial solutions common to all solutions In Proc. WABI’16, Workshop on Algorithms in Bioinformatics (ed. M. Frith and C.N.S. Pedersen), LNBI 9838, Springer, 2016, xiv, short abstract. 10.1109/TCBB.2017.2785831 29994355

